# Different percentages of metal doping in lithium titanate for the enhancement of electrochemical water splitting

**DOI:** 10.1039/d6ra06927d

**Published:** 2026-10-26

**Authors:** Md. Sahadat Hossain, Rifat Abedin, Umme Sarmeen Akhtar, Samina Ahmed

**Affiliations:** a Institute of Glass & Ceramic Research and Testing, Bangladesh Council of Scientific and Industrial Research (BCSIR) Dhaka 1205 Bangladesh saz8455@gmail.com; b Department of Applied Chemistry and Chemical Engineering, Faculty of Engineering and Technology, University of Dhaka Dhaka 1000 Bangladesh

## Abstract

Lithium titanate (Li_4_Ti_5_O_12_) is widely studied as an anode material in lithium-ion batteries because of its high structural stability, rapid lithium-ion transport, and superior safety. Due to its high bandgap energy, poor electrical conductivity, and poor diffusion kinetics, lithium titanate (Li_4_Ti_5_O_12_) is generally not considered a primary electrocatalyst. In this study, lithium titanate doped with different molar ratios of sodium was synthesized by a solid-state reaction in order to improve its electrocatalytic efficiency. The synthesized samples were characterized using X-ray diffraction (XRD), Fourier transform infrared spectroscopy (FTIR), field-emission scanning electron microscopy (SEM), UV/Vis/NIR spectroscopy and X-ray photoelectron spectroscopy (XPS). Additionally, electrocatalytic measurements confirmed that the biphasic Na_*x*_Li_4−*x*_Ti_5_O_12_ can successfully perform both hydrogen evolution reaction (HER) and oxygen evolution reaction (OER). Among the synthesized samples, Na_0.8_Li_3.2_Ti_5_O_12_ demonstrated the best catalytic activity for HER (−0.66 V *vs.* RHE at −10 mA cm^−2^) and onset potential of ∼0.15 V *vs.* RHE (at −1 mA cm^−2^). Na_0.6_Li_3.4_Ti_5_O_12_ exhibited superior electrocatalytic efficiency for OER (1.64 V *vs.* RHE at 10 mA cm^−2^) and an onset potential of ∼1.06 V *vs.* RHE (at 1 mA cm^−2^). This bifunctional catalytic activity of the sample makes it a promising and cost-effective alternative to expensive platinum-group metals for industrial electrocatalytic applications.

## Abstract 2

Lithium titanate (Li_4_Ti_5_O_12_) is used here for electrochemical water splitting and efficiency was increased by sodium doping.

## Graphical abstract

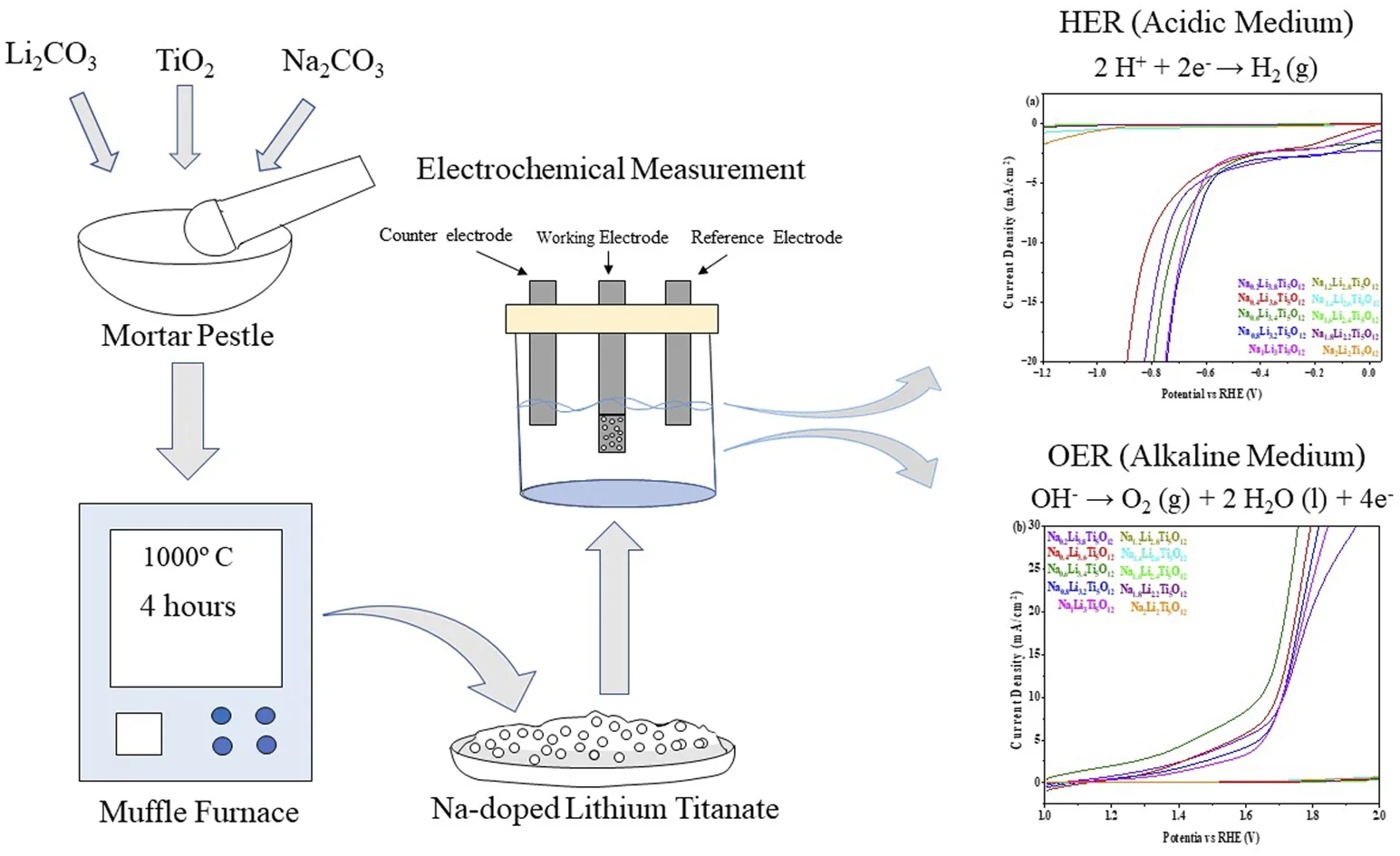

## 1 Introduction

Sustainable and green energy resources are becoming very important because pollution is increasing and fossil fuels are being consumed at an increasing rate. Recent studies focus on developing clean and sustainable energy solutions to solve current energy and environmental problems.^1,2^ Electrochemical water splitting is considered a promising method for producing clean and renewable energy, as it involves hydrogen production at the cathode (HER) and oxygen production at the anode (OER).^3,4^ In water splitting, the main challenge is the need for additional voltage, called overpotential, for both the hydrogen evolution reaction (HER) and the oxygen evolution reaction (OER). Under ideal equilibrium conditions, HER does not require additional voltage to proceed, but in real systems additional potential is required because of resistance from the electrolyte and the electrode–electrolyte interface. In contrast, OER requires a minimum voltage of 1.23 V even at equilibrium, along with an additional overpotential to drive the reaction.^5,6^ Usually, precious metal-based electrocatalysts such as Pt for HER and IrO_2_ for OER are regarded as standard catalysts. However, their high cost, limited availability, and poor stability have restricted their large-scale practical use.^7–9^ Therefore, electrochemical water splitting needs an efficient bifunctional electrocatalyst that can lower the required overpotential for both HER and OER.^5,10^

Several methods are used to improve the overall performance of electrocatalysts such as defect engineering, heterostructure formation, heteroatom doping, nanostructuring, and electrolyte tuning.^11–13^ Heteroatom doping is widely used technique that changes the electronic properties of a material by redistributing charges within it. An important benefit of this method is that it can retain the basic structure of the material, so its fundamental properties remain the same. Heteroatom doping has been widely used in carbon materials and metal compounds and has proven effective in improving their electrocatalytic performance.^14,15^ Sodium doping is one of the heteroatom doping techniques that has been used to improve both HER and OER. Na doping boosts the HER activity of NiS by improving electrical conductivity and adjusting hydrogen adsorption, which results in lower overpotential and faster reaction kinetics.^16^ For Na-doped ruthenium perovskite, Na doping reduces the adsorption energies by shifting the surface oxygen p-band and the Ru d-band.^17^ In Na-doped Co_3_O_4_ spinel, Na lowers the bandgap for better conductivity and reduces the *O to *OOH free energy barrier, yielding low overpotentials.^18^

Spinel lithium titanate (Li_4_Ti_5_O_12_) has gained increasing interest as an electrode material in lithium-ion batteries due to its zero-strain structure, excellent cycling stability, and high safety.^19,20^ Compared with graphite, Li_4_Ti_5_O_12_ has higher lithium-ion mobility, which makes it suitable for high-rate battery performance.^21^ Moreover, lithium titanate (Li_4_Ti_5_O_12_) is non-toxic, low-cost, and performs well even at low temperatures, showing highly reversible lithium-ion reactions. It also undergoes very little volume change during lithium-ion insertion and removal, which gives it excellent cycle stability and reduces the risk of dendrite formation, a problem often seen in carbon-based anodes.^22^ However, the major limitation of lithium titanate is its extremely low electrical conductivity (∼10^−13^ S cm^−1^) and ionic conductivity (10^−13^–10^−9^ cm^2^ s^−1^). To overcome these limitations, several strategies have been developed, such as reducing the particle size of LTO to shorten the diffusion path of Li^+^ ions and electrons, introducing ion doping, and adding conductive nanomaterials.^23^ Among these approaches, metal ion doping is considered one of the most effective strategies for improving the electrochemical performance of lithium titanate. Cr-doped lithium titanate has demonstrated improved cycling stability and higher reversible capacity compared with pristine lithium titanate.^24^ Mg/La co-doping modifies the electronic density of states and reduces the Li^+^ diffusion barrier.^25^ Although lithium titanate (Li_4_Ti_5_O_12_) has been widely studied as an electrode material, studies focusing on its electrocatalytic applications are very limited.

In this study, Na-doped lithium titanate samples were synthesized by a solid-state method. Different techniques were used to characterize the synthesized samples and investigate the electrocatalytic properties through HER and OER. Lithium titanate was doped with different molar ratios of sodium, and the change in catalytic performance due to variations in sodium doping were evaluated. This study focused on understanding how sodium doping changes the properties of lithium titanate (LTO) and improves its performance in both HER and OER. Furthermore, the reaction mechanisms were discussed.

## 2 Materials and methods

### 2.1 Materials

Sodium carbonate (Na_2_CO_3_) (purity 99%) was purchased from VWR Chemicals BDH. Titanium dioxide (TiO_2_) (purity 99.8%), lithium carbonate (Li_2_CO_3_) (purity >99%), sulfuric acid (H_2_SO_4_) (Purity 98%) and potassium hydroxide (KOH) (Purity ≥85%) were purchased from Merck, Germany. All the chemicals were used without any further purification. Deionized water obtained *via* double distillation was employed to avoid the presence of ionic impurities.

### 2.2 Methods

#### 2.2.1 Synthesis of lithium titanate and Na-doped lithium titanate

Pristine lithium titanate (LTO) was synthesized by a solid-state method. In this method, the Li_2_CO_3_ and TiO_2_ were mixed in such a way to obtain a lithium-to-titanium molar ratio of 4 : 5 (in accordance with the stoichiometry in Li_4_Ti_5_O_12_). However, an excess Li_2_CO_3_ was added to compensate the loss of lithium through volatilization during high temperature calcination. Subsequently, the mixture was ground using a mortar and pestle for ∼30 min. The obtained homogeneous mixture was calcined at 1000 °C for 4 hours in a muffle furnace and then naturally cooled to room temperature. The Na-doped lithium titanate samples were also prepared using a similar method by mixing Li_2_CO_3_, Na_2_CO_3_ and TiO_2_, where the nominal molar ratios of Lithium, sodium and titanium were 4 − *x*: *x*: 5 (*x* = 0.2, 0.4, 0.6, 0.8, 1, 1.2, 1.4, 1.6, 1.8, and 2). Accordingly, the samples are denoted as Na_*x*_Li_4−*x*_Ti_5_O_12_, where *x* represents the nominal Na doping level used during synthesis.

### 2.3 Characterization

The phase analysis of the synthesized pristine LTO and Na-doped lithium titanate (Na_*x*_Li_4−*x*_Ti_5_O_12_) was carried out using a Rigaku SE X-ray diffraction instrument. A Cu Kα radiation source was employed with an operating voltage of 40 kV and a current of 50 mA to generate the characteristic X-rays, using a scanning rate of 35° min^−1^. The diffraction patterns were recorded over a 2*θ* range of 15–70° with a step size of 0.01°, following the Bragg–Brentano geometry. An IR-Prestige 21 spectrometer (Shimadzu, Japan) equipped with an attenuated total reflection (ATR) accessory was used to investigate the functional properties of pristine LTO. The infrared spectrum was recorded over a wavenumber range of 400–4000 cm^−1^, with the data obtained from an average of 30 scans at a spectral resolution of 4 cm^−1^. In addition, the surface morphology of the pristine LTO was observed with the help of an energy-dispersive X-ray (EDX)-equipped FESEM (JSM-7610F). All the images were collected at an accelerating voltage of 25 kV. The sample was sputter-coated with gold and dried, after which it was dispersed in ethanol prior to analysis. Moreover, an XPS instrument (Thermo Scientific K-Alpha) was used to analyze the chemical state of the synthesized pristine LTO. Furthermore, a PerkinElmer LAMBDA 1050+ UV-Vis-NIR spectrophotometer was used to investigate the optical properties of the samples. The measurements were performed in diffuse reflectance mode using powdered samples over a wavelength range of 200–600 nm.

### 2.4 Electrocatalytic assessment

Electrochemical measurements were performed using a CS300 (Corrtest, China) with a three-electrode system, where Pt, Ag/AgCl, and a modified glassy carbon (GC) electrode served as the counter, reference, and working electrodes, respectively. The GC electrode was modified each time using the synthesized Na_*x*_Li_4−*x*_Ti_5_O_12_ sample with *x* = 0.2, 0.4, 0.6, 0.8, 1, 1.2, 1.4, 1.6, 1.8, and 2. For working electrode preparation, 10 mg of the catalyst was dispersed in 1.5 mL of acetone and sonicated for 30 min to obtain a uniform catalyst suspension. Subsequently, 10 µL of the prepared ink was drop-cast onto a glassy carbon electrode with a geometric area of 0.12 cm^2^ and allowed to dry gradually at room temperature. The resulting catalyst loading on the working electrode was 0.56 mg cm^−2^. The electrocatalytic activities of the Na_*x*_Li_4−*x*_Ti_5_O_12_ samples were evaluated in an aqueous H_2_SO_4_ solution (0.5 M) for HER and in an aqueous KOH solution (0.5 M) for OER using the linear sweep voltammetry (LSV) at 25 °C with a scan rate of 25 mV s^−1^. The polarization curves were not subjected to *iR* correction. The potentials were converted to the reversible hydrogen electrode (RHE) according to the following eqn (1):

1 *E*_RHE_ = *E*_Ag/AgCl_ + 0.197 + 0.0591 × pH

And the overpotentials (*η*) for HER and OER were calculated according to the eqn (2) and (3):

2 *η*_HER_ = *E*_RHE_

3 *η*_OER_ = *E*_RHE_ − 1.23 V

## 3 Results

### 3.1 X-ray diffraction analysis

Fig. 1a illustrates the X-ray diffractions patterns of the pristine LTO and Na_*x*_Li_4−*x*_Ti_5_O_12_, where *x* denotes 0.2, 0.4, 0.6, 0.8, 1, 1.2, 1.4, 1.6, 1.8, and 2. All the Na_*x*_Li_4−*x*_Ti_5_O_12_ samples exhibited characteristic peaks at *d*-spacing (measured in Å) corresponding to the Miller indices of 4.83 (111), 2.52 (311), 2.09 (400), 1.91 (331), 1.60 (333), 1.47 (440) and 1.41 (531). The data obtained showed good agreement with the standard ICDD database (card no. 00-049-0207) for LTO, thereby validating the presence of a crystalline spinel structure (space group: *Fd*3̄*m* (227)).^26^ However, the characteristic (111) peak of LTO shifted toward a lower 2*θ* value, as shown in Fig. 1b, indicating an increase in the lattice parameter due to the replacement of Li^+^ with Na^+^ in the spinel structure.^27^ Interestingly, the peak shift was small, suggesting that only a limited amount of Na^+^ was incorporated into the LTO lattice. In addition to the LTO peaks, a new diffraction peak at 2*θ* ≈ 15.9° was observed, which was assigned to the crystallographic plane (410) of the monoclinic structure (*P*2_1_/*m*) of disodium trititanate (Na_2_Ti_3_O_7_).^28^ This peak was noticeable beyond a certain doping level. Moreover, when the sodium content was increased in the samples, the intensity of the characteristic peak of LTO (*d* = 4.83) gradually decreased (as illustrated in Fig. 1c). The appearance and progressive increase in the intensity of this new peak with increasing Na content indicate the formation of a secondary Na_2_Ti_3_O_7_ phase. To quantitatively evaluate the phase evolution, Rietveld refinement was performed on the XRD patterns, and the corresponding phase fractions are presented in Table 1, while the Rietveld-refined XRD profiles of Na_0.2_Li_3.8_Ti_5_O_12_, Na_0.6_Li_3.4_Ti_5_O_12_, Na_0.8_Li_3.2_Ti_5_O_12_ and Na_2_Li_2_Ti_5_O_12_ are presented in Fig. 2. The refinement confirmed that LTO remained the major crystalline phase throughout the investigated composition range. For *x* = 0.2–1.0, the LTO phase accounted for 98.7–91.8 wt%, while the Na_2_Ti_3_O_7_ phase remained relatively minor (1.3–8.2 wt%). However, a significant increase in the Na_2_Ti_3_O_7_ fraction was observed when the nominal Na content exceeded *x* = 1.0, increasing from 19.8 wt% at *x* = 1.2 to 37.2 wt% at *x* = 2.0. These results indicate that, although the LTO structure remains the predominant phase, its ability to accommodate additional Na is increasingly limited at higher nominal Na contents. Consequently, beyond *x* = 1.0, an increasing fraction of the added Na likely contributes to the formation of the Na_2_Ti_3_O_7_ secondary phase rather than being incorporated solely into the LTO lattice. Therefore, the *x* values used in this study represent the nominal Na contents of the starting compositions, rather than the actual amount of Na incorporated into the LTO lattice. As LTO was the primary phase of interest in the biphasic Na_*x*_Li_4−*x*_Ti_5_O_12_, and since the disodium trititanate phase started to form during Na doping, SEM, bandgap calculation, and XPS analysis were carried out only for pristine LTO to identify the structural parameters of the pure spinel phase.

**Fig. 1:**
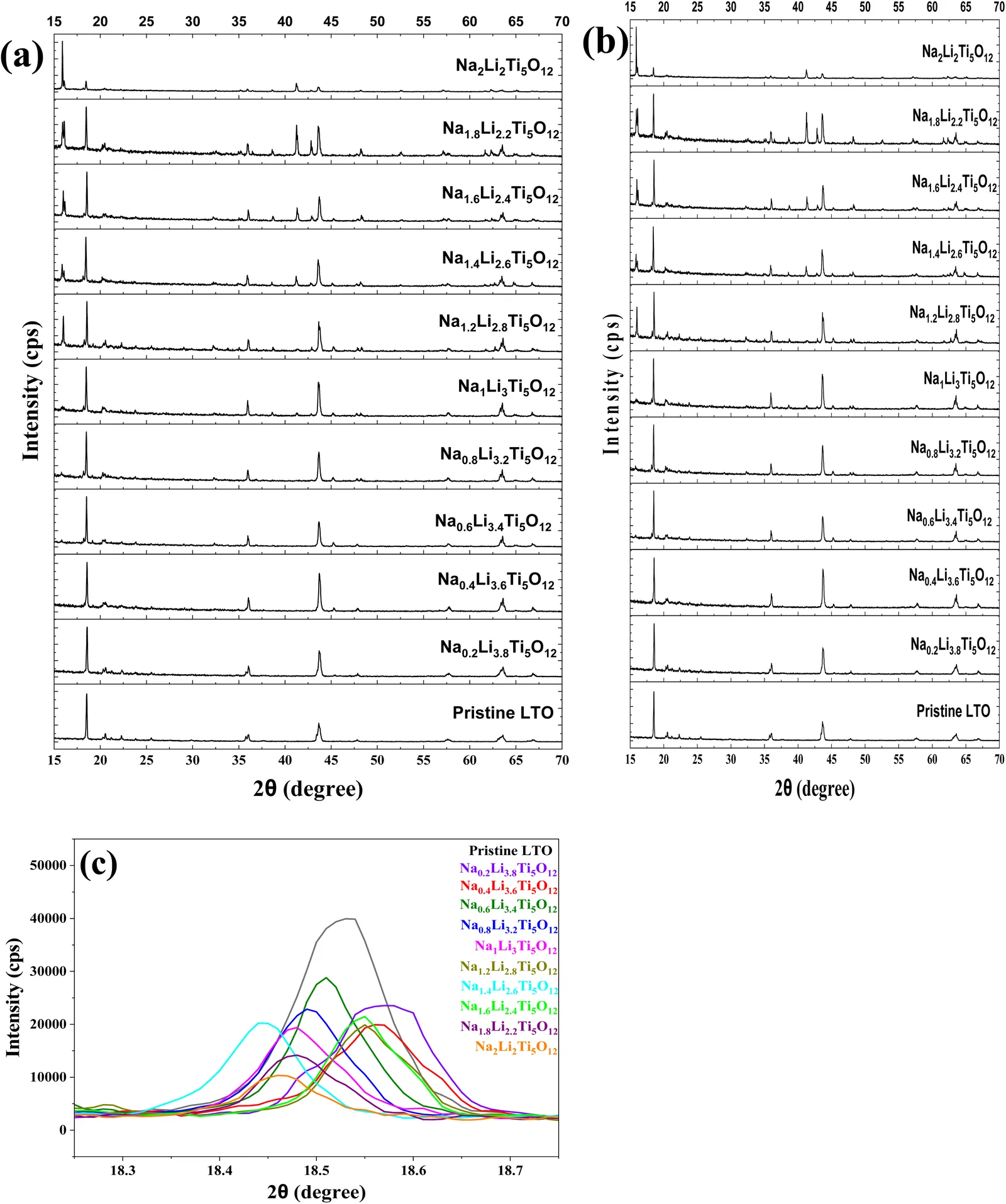
XRD patterns of pristine LTO and Na_*x*_Li_4−*x*_Ti_5_O_12_ (a) full range 15°–70°, (b) selected range 18.25°–19° showing peak shifting, and (c) selected range 18.25°–18.75° showing intensity decrease.

**Table 1:** Phase weight fractions of the prepared samples determined by Rietveld analysis

Sample	Nominal Na content (*x*)	Li_4_Ti_5_O_12_ (wt%)	Na_2_Ti_3_O_7_ (wt%)
Na_0.2_Li_3.8_Ti_5_O_12_	0.2	98.7	1.3
Na_0.4_Li_3.6_Ti_5_O_12_	0.4	95.3	4.7
Na_0.6_Li_3.4_Ti_5_O_12_	0.6	94	6
Na_0.8_Li_3.2_Ti_5_O_12_	0.8	92.9	7.1
Na_1_Li_3_Ti_5_O_12_	1	91.8	8.2
Na_1.2_Li_2.8_Ti_5_O_12_	1.2	80.2	19.8
Na_1.4_Li_2.6_Ti_5_O_12_	1.4	78.7	21.3
Na_1.6_Li_2.4_Ti_5_O_12_	1.6	74.4	25.6
Na_1.8_Li_2.2_Ti_5_O_12_	1.8	69.3	30.7
Na_2_Li_2_Ti_5_O_12_	2	62.8	37.2

**Fig. 2:**
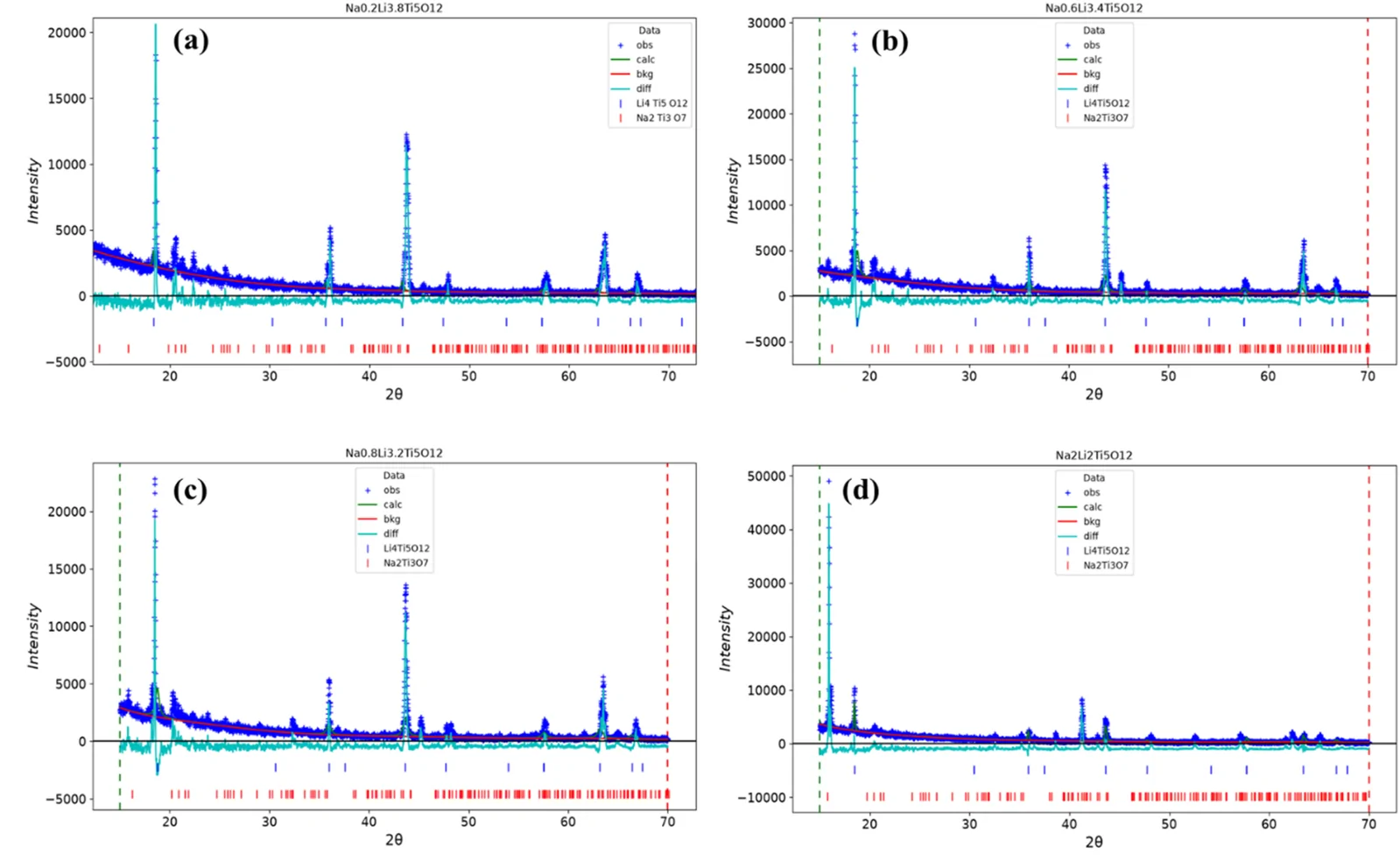
Rietveld-refined XRD patterns of (a) Na_0.2_Li_3.8_Ti_5_O_12_, (b) Na_0.6_Li_3.4_Ti_5_O_12_, (c) Na_0.8_Li_3.2_Ti_5_O_12_, and (d) Na_2_Li_2_Ti_5_O_12_.

### 3.2 FTIR analysis

The Fourier Transform Infrared (FTIR) spectra of pristine LTO, Na_0.6_Li_3.4_Ti_5_O_12_ and Na_0.8_Li_3.2_Ti_5_O_12_ were recorded to identify the functional groups and is presented in Fig. 3. For pristine LTO, a peak appearing in the range of 625–630 cm^−1^ was observed, which corresponds to the symmetric stretching vibration of the Ti–O bond.^29^ Additionally, Li–O stretching vibration bands were detected in the region between ∼500 and ∼675 cm^−1^.^30^ The presence of spinel-related vibrations at low wavenumbers, in the range from 400 to 700 cm^−1^, confirms the formation of LTO. In particular, the low-frequency bands centered at ∼464 and ∼651 cm^−1^ are assigned to the asymmetric and symmetric stretching modes of the TiO_6_ octahedra, respectively.^31^ For Na_0.6_Li_3.4_Ti_5_O_12_ and Na_0.8_Li_3.2_Ti_5_O_12_, a new peak appeared at 1437 cm^−1^, which was assigned to the Na–O bond.^32^ Moreover, all the characteristic peaks of pristine LTO were retained in the spectra of Na_0.6_Li_3.4_Ti_5_O_12_ and Na_0.8_Li_3.2_Ti_5_O_12_, suggesting the preservation of the spinel LTO structure and supporting the incorporation of Na into the LTO structure.

**Fig. 3:**
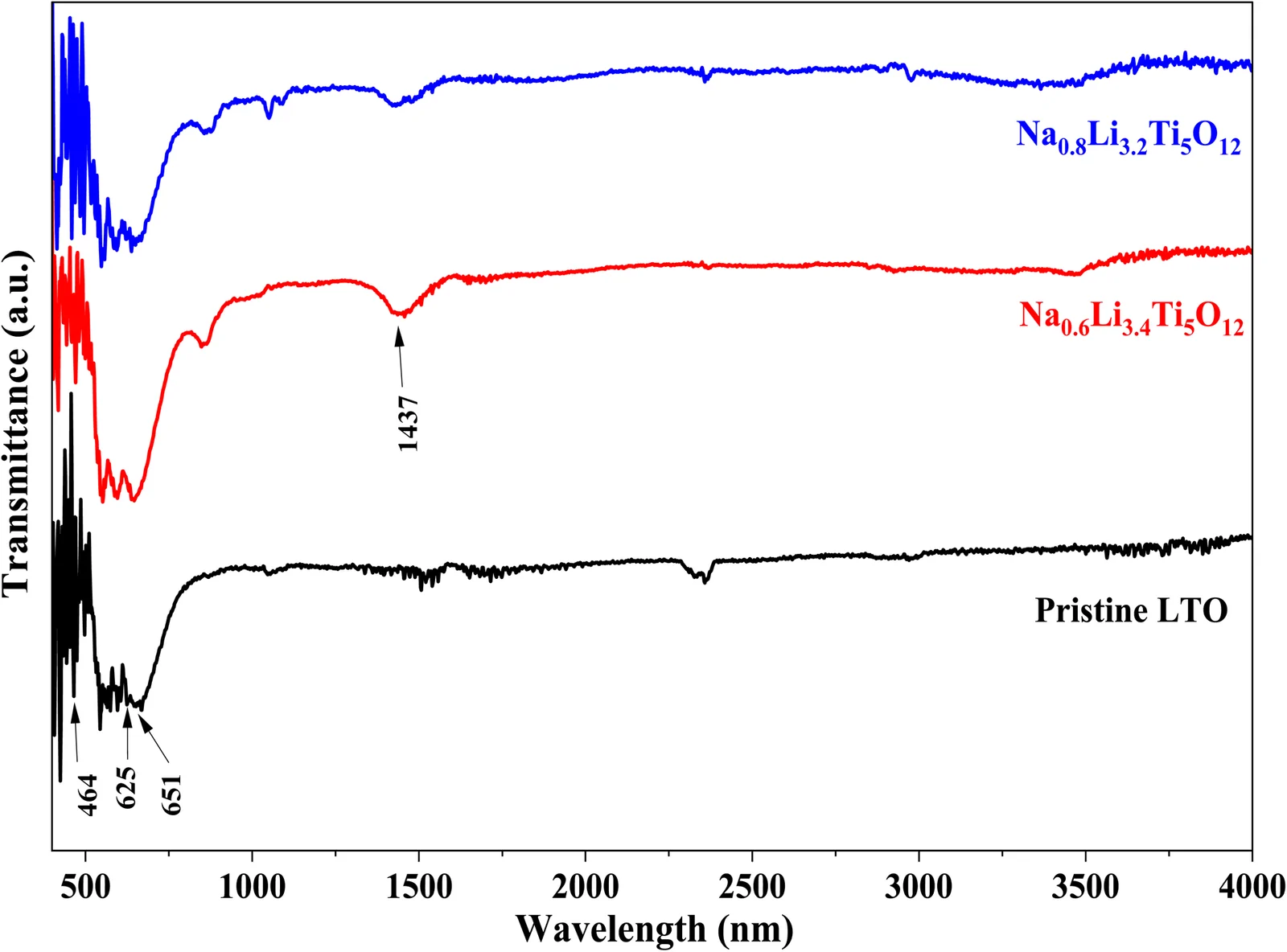
FTIR spectrum of pristine LTO, Na_0.6_Li_3.4_Ti_5_O_12_ and Na_0.8_Li_3.2_Ti_5_O_12_ in the full range of 400–4000 cm^−1^ wavenumber.

### 3.3 SEM analysis

Fig. 4 displays the SEM images of pristine LTO. It is clearly observed that the particles appear crystalline with clear and well-defined edges and exhibit a wide size distribution. Most particles are larger than 1 µm, while a smaller fraction is below 1 µm. Interestingly, the particles are agglomerated with each other. This agglomeration results in the formation of larger particle clusters (greater than 1 µm).^33^ Moreover, the particles are non-uniform in shape and their surfaces are rough and irregular.

**Fig. 4:**
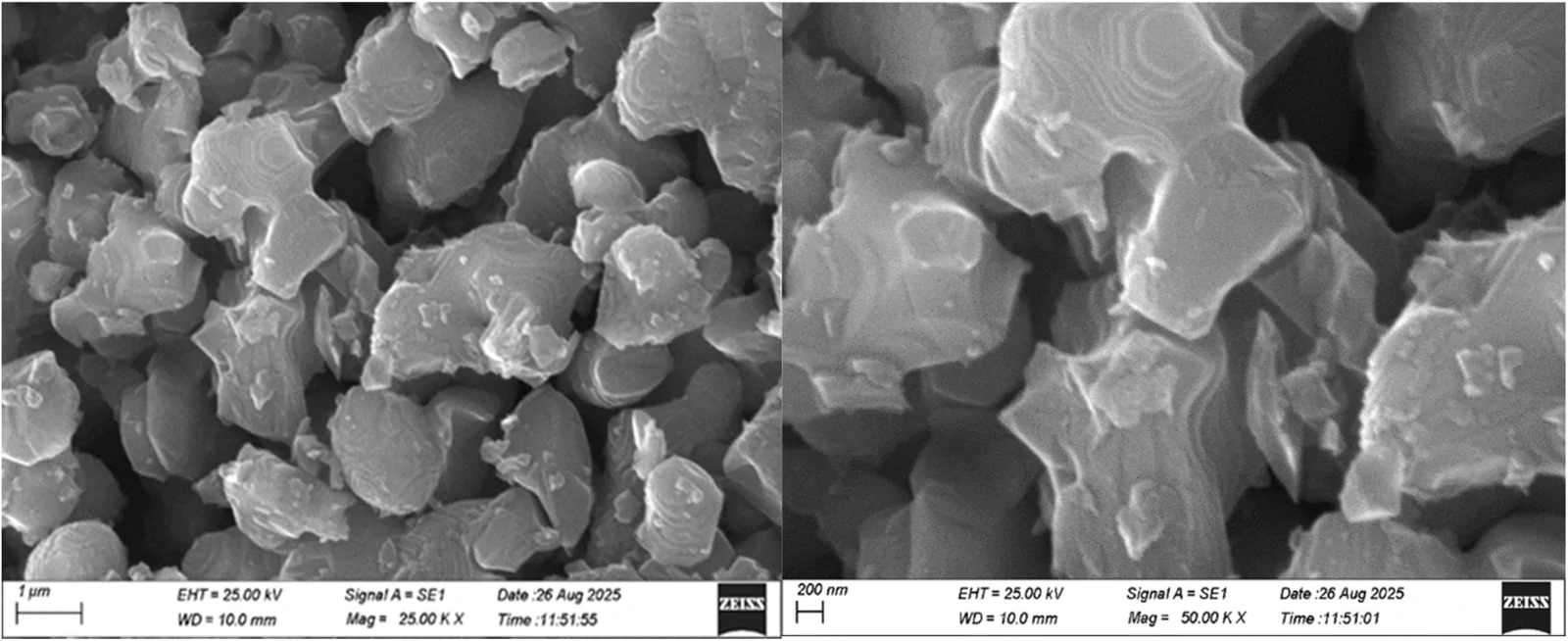
SEM images of pristine LTO.

### 3.4 Bandgap estimation

The diffuse reflectance spectrum of pristine LTO obtained from the UV-Vis-NIR measurements is presented in Fig. 5a. The reflectance data were converted into the Kubelka–Munk function, *F*(*R*), and used to construct the corresponding Tauc plot. The optical bandgap energy was estimated using eqn (4).^34^ The obtained optical bandgap for pristine LTO was 3.81 eV, as illustrated in Fig. 5b which is consistent with previously reported values.^35^ Due to its wide bandgap, LTO exhibits poor electronic conductivity. This behavior can be attributed to the electronic configuration of Ti^4+^, in which the t_2g_ orbitals are completely filled while the e_g_ orbitals remain empty, thereby limiting electron mobility.^36^

**Fig. 5:**
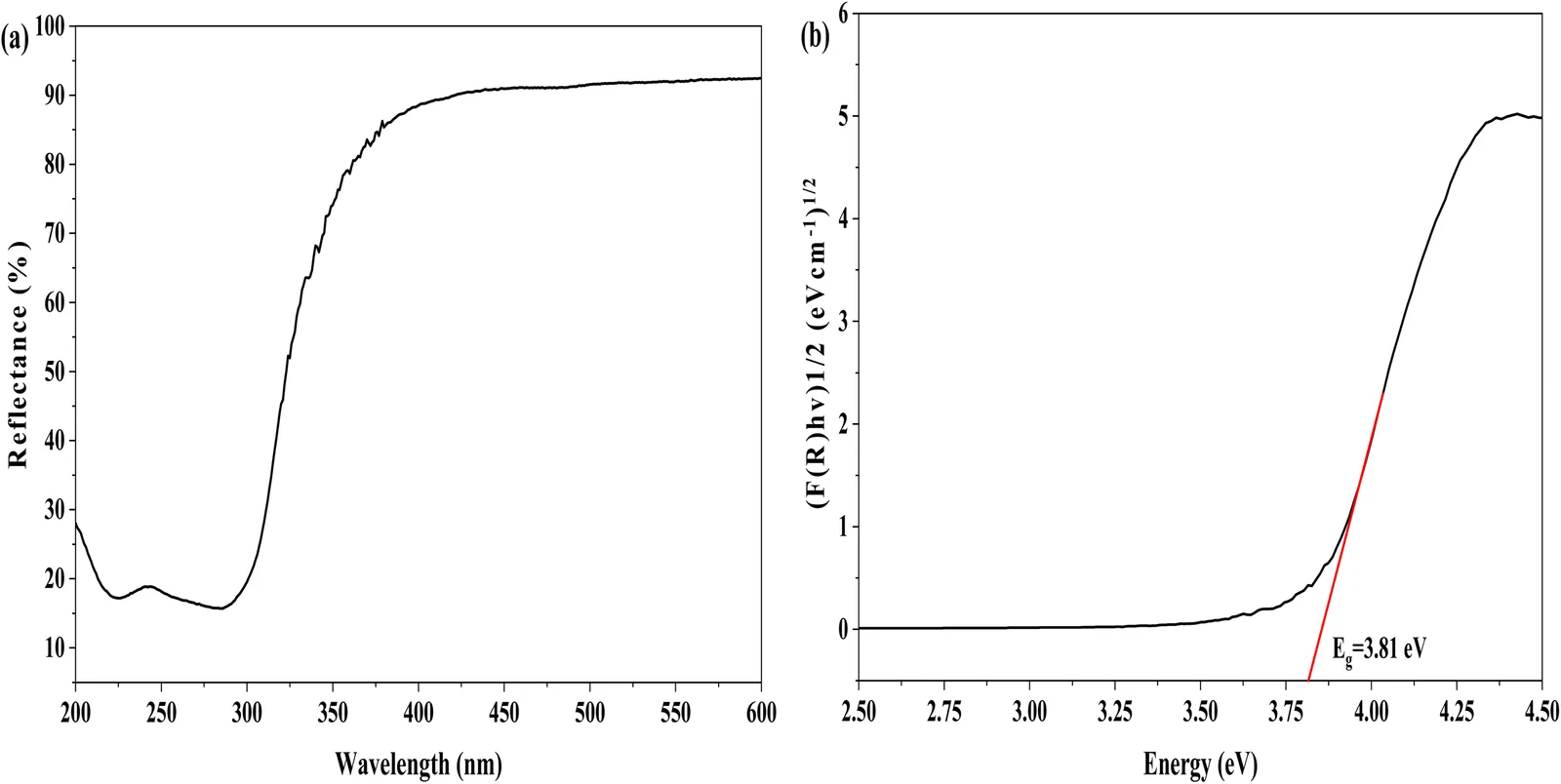
(a) Reflectance, and (b) indirect Tauc plot of pristine LTO.

Indirect Tauc plot:

4 (*F*(*R*)*hv*)^1/2^ = *A*(*hv* − *E*_g_)

where *F*(*R*) is a function of reflectance, *hv* is photon energy, *A* is constant, *E*_g_ is optical band gap.

### 3.5 XPS analysis

Fig. 6a represents the XPS wide scan spectrum of pristine LTO. The spectrum consists of several prominent peaks corresponding to lithium, titanium, oxygen, and carbon, and shows good agreement with previously reported literature.^37,38^Fig. 6b–e illustrate the spectra of Ti 2p, Li 1s, O 1s and C 1s of pristine LTO respectively. Fig. 6b reveals two dominant peaks at approximately 458.8 eV (Ti 2p_3/2_) and 464.4 eV (Ti 2p_1/2_), confirming the presence of Ti in the Ti^4+^ oxidation state in the LTO structure.^39,40^ The Li 1s XPS spectrum shown in Fig. 6c exhibits a weak single peak at 54.3 eV, which corresponds well to the binding energy of the Li–O bond in the spinel LTO structure.^41^Fig. 6d presents the O 1s spectrum, which includes a peak at around 529.6 eV attributed to O^2−^ in the O–Ti–O lattice, and another peak at approximately 531.1 eV associated with oxygen vacancies on the material surface.^42,43^ Moreover, Fig. 6e shows the binding enrgies of C 1s at ∼284.8, 286.5, and 289.8 eV, corresponding to the C–C, C–O and O–C=O bonds, respectively. These peaks indicate different types of carbon bonding present in the sample, probably caused by surface contamination.^44^

**Fig. 6:**
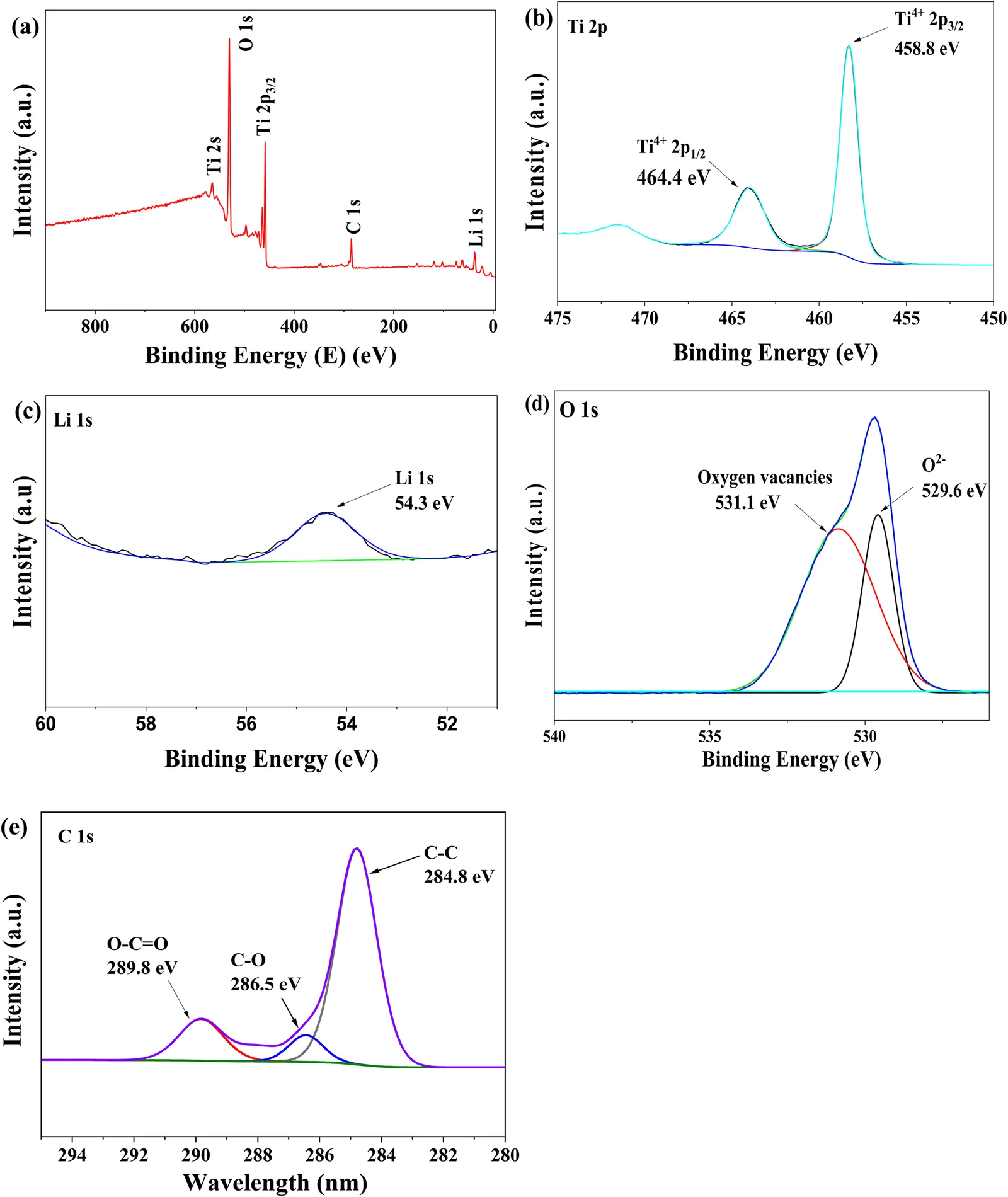
XPS analysis of pristine LTO (a) wide scan spectrum and narrow scan spectrum of (b) Ti 2p, (c) Li 1s, (d) O 1s, and (e) C 1s.

### 3.6 Electrocatalytic performance

The HER polarization curves and the OER polarization curves were obtained from linear sweep voltammetry (LSV) measurements. The HER polarization curves and the OER polarization curves of pristine LTO and Na_*x*_Li_4−*x*_Ti_5_O_12_ are shown in Fig. 7a and b, respectively. Compared with pristine LTO, Na incorporation enhanced the HER and OER activities when the nominal Na content was maintained at or below *x* = 1.0. Interestingly, the biphasic samples showed better HER performance than OER activity because the required potential *vs.* RHE for HER was lower than that for OER. In Fig. 7a, Na_0.8_Li_3.2_Ti_5_O_12_ exhibited the best HER electrocatalytic performance, which required approximately −0.66 V *vs.* RHE to achieve a current density of −10 mA cm^−2^, a commonly used benchmark current density for evaluating electrocatalytic HER performance. This was a lower value than those of Na_1_Li_3_Ti_5_O_12_ (−0.68 V *vs.* RHE), Na_0.6_Li_3.4_Ti_5_O_12_ (−0.71 V *vs.* RHE), Na_0.2_Li_3.8_Ti_5_O_12_ (−0.75 V *vs.* RHE), and Na_0.4_Li_3.6_Ti_5_O_12_ (−0.81 V *vs.* RHE). Na incorporation clearly improved the HER and OER activity of the samples. Furthermore, Na_0.8_Li_3.8_Ti_5_O_12_ showed an onset potential of ∼0.15 V *vs.* RHE (at −1 mA cm^−2^) for HER, suggesting the potential at which the hydrogen evolution reaction becomes kinetically favorable.^45^ The HER reactions that were involved are presented in eqn (5)–(7):^46^

5 H^+^ + e^−^ → H* (Volmer)

6 H^+^ + e^−^ + H* → H_2_ (Heyrovsky)

or

7 2H* → H_2_ (Tafel)

where * refers to an active site on the surface of the electrocatalyst.

**Fig. 7:**
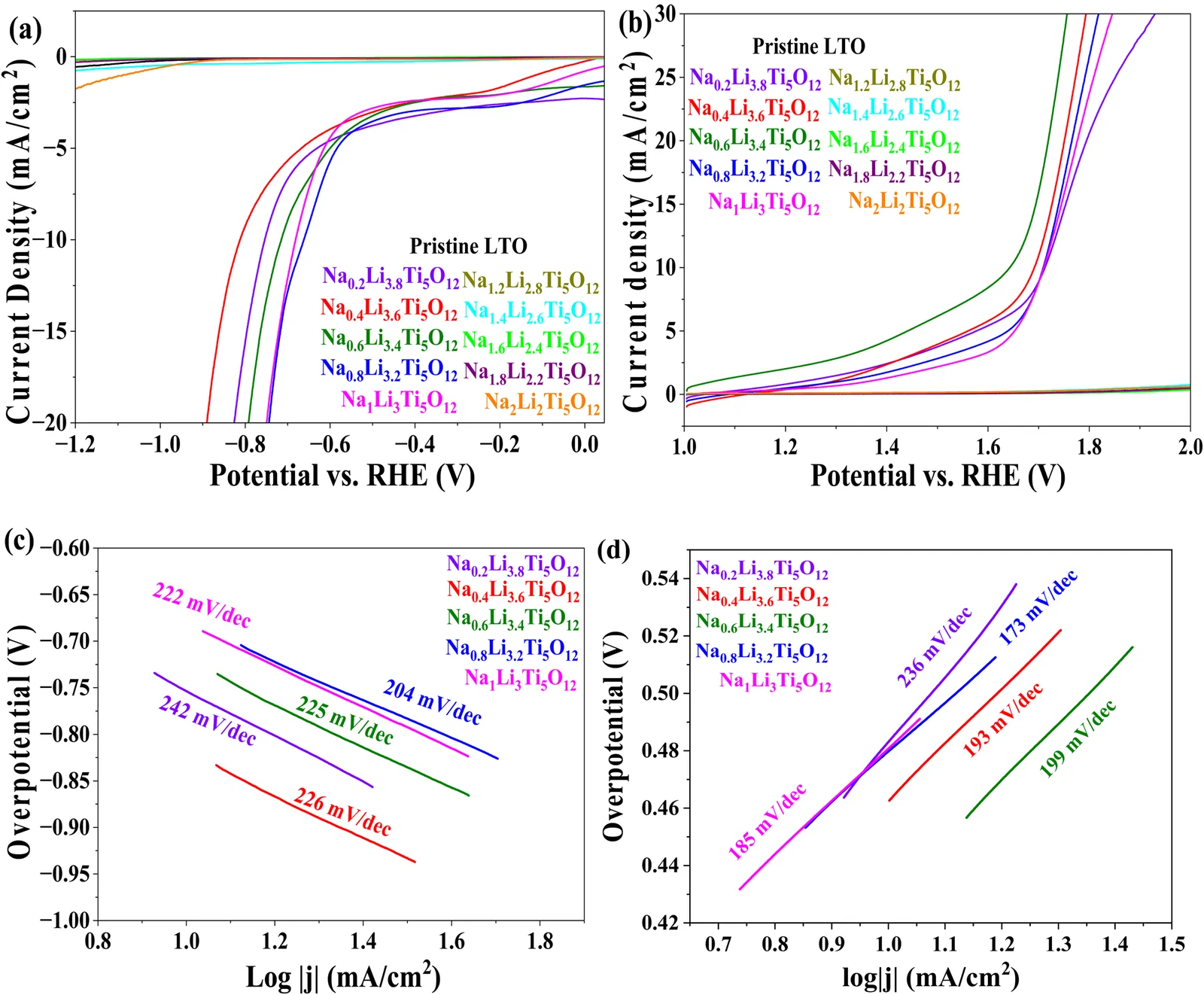
The polarization curve of pristine LTO and Na_*x*_Li_4−*x*_Ti_5_O_12_ for (a) HER, (b) OER and the Tafel plot of Na_*x*_Li_4−*x*_Ti_5_O_12_ for (c) HER, (d) OER.

The first step in the multi-step HER process was the adsorption of hydrogen on the active site on the electrode surface when protons were discharged. This step was known as the Volmer reaction. In the next step, hydrogen gas (H_2_) was released from the cathode either through an electrochemical process called the Heyrovsky step or through a chemical process known as the Tafel step.^46^

On the other hand, Na_0.6_Li_3.4_Ti_5_O_12_ required the lowest potential *vs.* RHE to reach a current density of 10 mA cm^−2^ for OER in Fig. 7b, which was ∼1.64 V *vs.* RHE. Meanwhile, the potential *vs.* RHE values for Na_0.4_Li_3.6_Ti_5_O_12_, Na_0.2_Li_3.8_Ti_5_O_12_, Na_0.8_Li_3.2_Ti_5_O_12_, and Na_1_Li_3_Ti_5_O_12_ for OER were ∼1.69, 1.71, 1.71, and 1.71 V *vs.* RHE, respectively. Moreover, the onset potential of Na_0.6_Li_3.4_Ti_5_O_12_ for OER was ∼1.06 V *vs.* RHE (at 1 mA cm^−2^), indicating the point where oxygen generation began to proceed efficiently.^45^ The oxygen evolution reaction (OER) proceeded through a four-electron transfer pathway, as described in eqn (8)–(11):^45^

8 * + OH^−^ → OH* + e^−^

9 OH* + OH^−^ → O* + H_2_O + e^−^

10 O* + OH^−^ → OOH* + e^−^

11 OOH* + OH^−^ → O_2_ + H_2_O + e^−^

Here, * indicates the active site where the OER takes place. The species OH*, O*, and OOH* represent intermediate species that were temporarily adsorbed on these active sites.

The Tafel slope is an intrinsic characteristic of a catalyst and depends on the rate-determining step of the HER and OER. Therefore, analyzing the Tafel slope is important for understanding the basic reaction steps involved in the process.^47^ It can be determined by plotting overpotential (*η*) against log |*j*| using the equation *η* = *b* log(*j*/*j*_0_), where *j* is the current density and *j*_0_ is the exchange current density. The value of *b* is obtained from the linear region of the Tafel plot.^48^ In theory at 25 °C, the Tafel slope is expected to be about ∼120 mV dec^−1^ when the Volmer step is the rate-determining step. However, Tafel slope values of around ∼40 mV dec^−1^ and ∼30 mV dec^−1^ are generally associated with the Heyrovsky step and the Tafel step, respectively.^49^Fig. 7c and d display the Tafel plots of Na_*x*_Li_4−*x*_Ti_5_O_12_ for HER and OER, respectively. The Tafel slope value of Na_0.8_Li_3.2_Ti_5_O_12_ for HER (Fig. 7c) was 204 mV dec^−1^, which was lower than Na_1_Li_3_Ti_5_O_12_ (222 mV dec^−1^), Na_0.6_Li_3.4_Ti_5_O_12_ (225 mV dec^−1^), Na_0.4_Li_3.6_Ti_5_O_12_ (226 mV dec^−1^), and Na_0.2_Li_3.8_Ti_5_O_12_ (242 mV dec^−1^). Relevant data are registered in Table 2. The experimentally obtained Tafel slopes were considerably higher than the theoretical value associated with the Volmer step. Therefore, the Tafel slopes alone cannot be used to reliably identify the rate-determining step. The lower Tafel slope of Na_0.8_Li_3.2_Ti_5_O_12_ compared with the other compositions indicates more favorable apparent HER kinetics. In case of OER, Na_0.8_Li_3.2_Ti_5_O_12_ had the lowest Tafel slope value of 173 mV dec^−1^. Moreover, the Tafel slope values of Na_1_Li_3_Ti_5_O_12_, Na_0.4_Li_3.6_Ti_5_O_12_, Na_0.6_Li_3.4_Ti_5_O_12_, and Na_0.2_Li_3.8_Ti_5_O_12_ for OER were 185, 193, 199 and 236 mV dec^−1^, respectively. The OER Tafel slopes were also considerably higher than the theoretical value of approximately 120 mV dec^−1^ associated with the initial electron-transfer step under ideal kinetic conditions. The OER Tafel slopes were also considerably higher than the theoretical value of approximately 120 mV dec^−1^ associated with the initial electron-transfer step under ideal kinetic conditions. Therefore, the Tafel slopes alone cannot be used to reliably identify the rate-determining step. The lower Tafel slope of Na_0.8_Li_3.2_Ti_5_O_12_ compared with the other compositions indicates more favorable apparent OER kinetics. The relatively lower Tafel slopes for OER compared to HER indicate more favorable electron transfer kinetics of Na_*x*_Li_4−*x*_Ti_5_O_12_ for the OER reaction.

**Table 2:** Electrochemical parameters of HER and OER

Composite	HER		OER	
	Potential *vs.* RHE at 10 mA cm^−2^ (V)	Tafel slope (mV dec^−1^)	Potential *vs.* RHE at 10 mA cm^−2^ (V)	Tafel slope (mV dec^−1^)
Na_0.2_Li_3.8_Ti_5_O_12_	−0.75	242	1.71	236
Na_0.4_Li_3.6_Ti_5_O_12_	−0.81	226	1.69	193
Na_0.6_Li_3.4_Ti_5_O_12_	−0.71	225	1.64	199
Na_0.8_Li_3.2_Ti_5_O_12_	−0.66	204	1.71	173
Na_1_Li_3_Ti_5_O_12_	−0.68	222	1.71	185

The stability of the optimized HER and OER samples was evaluated by chronoamperometry for 30 min. Na_0.8_Li_3.2_Ti_5_O_12_ and Na_0.6_Li_3.4_Ti_5_O_12_ were tested at −0.5 V and 1.4 V *vs.* RHE for HER and OER, respectively, as shown in Fig. 8a and b. The LSV curves recorded before and after the 30 min chronoamperometric test were also compared. Both Na_0.8_Li_3.2_Ti_5_O_12_ and Na_0.6_Li_3.4_Ti_5_O_12_ showed a substantial decrease in current response after the chronoamperometric test, indicating a considerable loss of electrocatalytic activity during the 30 min operation.

**Fig. 8:**
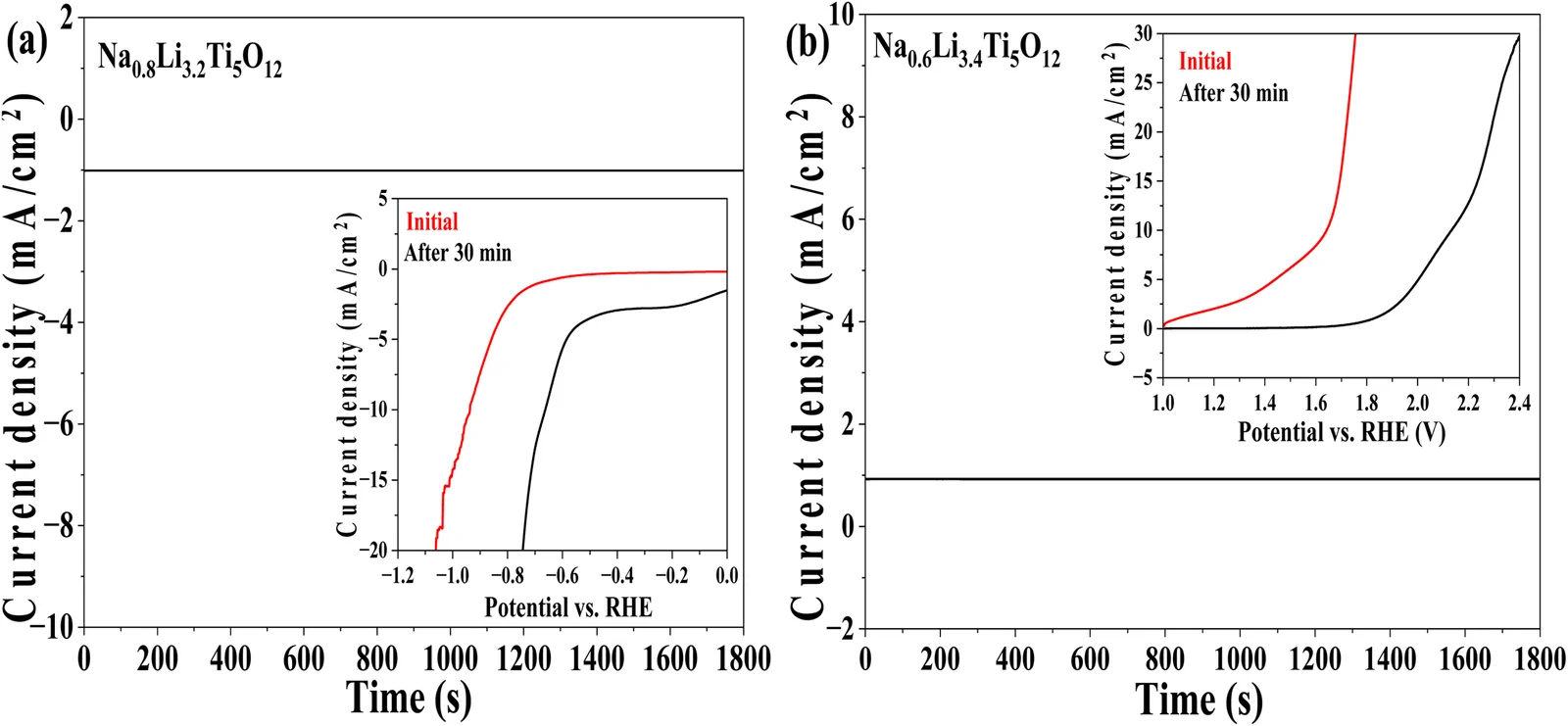
The chronoamperometry with initial and after 30 min LSV curves for (a) Na_0.8_Li_3.2_Ti_5_O_12_ at −0.5 V *vs.* RHE for HER, (b) Na_0.6_Li_3.4_Ti_5_O_12_ at 1.4 V *vs.* RHE for OER.

## 4 Discussion

The electrocatalytic performance indicates that Na-doping effectively improves the HER and OER activities of the biphasic Na_*x*_Li_4−*x*_Ti_5_O_12_, although the pristine LTO is not used as an electrocatalyst due to its sluggish ion diffusion kinetics, low electrical conductivity (10^−13^ S cm^−1^) and high bandgap energy.^23,50^ Firstly, Na-doping results in the larger Na^+^ ions (1.06 Å) occupying the sites of the smaller Li^+^ ions (0.76 Å) in the spinel structure, which enlarges the diffusion tunnels in the structure (consistent with the XRD data).^27^ Secondly, Na-doping may plausibly induce the conversion of Ti^4+^ to Ti^3+^ as a charge-compensation mechanism, potentially enhancing electronic conductivity and reducing the bandgap, consistent with previous reports on doped LTO systems.^51–53^ However, as Ti^3+^ formation was not directly verified in the present study, this mechanism should be regarded as a plausible interpretation rather than an experimentally confirmed phenomenon. As a result, biphasic Na_*x*_Li_4−*x*_Ti_5_O_12_ is capable of performing HER and OER at Na doping levels (from *x* = 0.2 to *x* = 1), as shown in Fig. 7a and b, respectively. Interestingly, at higher Na doping levels (from *x* = 1.2 to *x* = 2), the favorable potentials for both HER and OER become extremely high. The performance improves up to a certain Na doping level and then decreases due to the increasing fraction of the disodium trititanate phase. The increasing amount of the secondary Na_2_Ti_3_O_7_ phase may reduce the contribution of the LTO primary phase to the electrocatalytic activity, leading to higher potentials.

As the potentials for Na_*x*_Li_4−*x*_Ti_5_O_12_ (where *x* = 1.2–2) are very high, the Tafel slopes were only measured for the compositions with *x* = 0.2–1. The measured Tafel slope values for OER and HER were considerably higher than the theoretical value of approximately 120 mV dec^−1^. Such higher Tafel slopes than theoretical slope values may arise from non-kinetic behaviors, including bubble accumulation and mass-transport limitations.^54^ In addition, the absence of *iR* correction in the present measurements may have contributed to the relatively high apparent Tafel slopes. Therefore, the obtained Tafel slopes should not be used alone to conclusively identify the rate-determining steps.

## 5 Conclusion

In this study, lithium titanate was successfully doped with sodium which was confirmed from the XRD analysis. Na doping significantly improved the electrocatalytic performance of LTO toward both HER and OER. Na_0.8_Li_3.2_Ti_5_O_12_ displayed superior electrocatalytic properties for HER, achieving a current density of −10 mA cm^−2^ at −0.66 V *vs.* RHE, with a Tafel slope of 204 mV dec^−1^. For OER, Na_0.6_Li_3.4_Ti_5_O_12_ showed the best electrocatalytic performance, reaching a current density of 10 mA cm^−2^ at 1.64 V *vs.* RHE, with a Tafel slope of 199 mV dec^−1^. The relatively lower Tafel slopes of the Na-doped LTO samples indicate more favorable apparent reaction kinetics, although the Tafel slopes alone are not sufficient to identify the rate-determining steps. Although LTO is more commonly studied as a Li-ion battery anode, this work reveals the capability of LTO to utilize as a bifunctional electrocatalyst and as a low-cost alternative to conventional electrocatalysts. Next research will be based on developing different metal doped LTO electrocatalyst that has efficiency similar to the conventionally used electrocatalyst and optimized it.

## Author contributions

Md. Sahadat Hossain: conceived and designed the study; analyzed the data; supervised the overall work and reviewed the manuscript. Rifat Abedin: analyzed the data; wrote the manuscript, Umme Sarmeen Akhtar: data curation, Samina Ahmed: supervised the findings of this work.

## Conflicts of interest

There are no conflicts to declare.

## Data Availability

Data will be made available on request.
